# Cardiotoxicity of Chemoradiotherapy in Patients With Esophageal Cancer: A Systematic Review

**DOI:** 10.7759/cureus.100822

**Published:** 2026-01-05

**Authors:** Cyril Kocherry, Hina Shamim, Kiran Jhakri, Moath Al-Shudifat, Bushra Sumra, Ann Kashmer Yu

**Affiliations:** 1 School of Medicine, Ninewells Hospital, Dundee, GBR; 2 Pediatrics, Baqai Medical University, Karachi, PAK; 3 Internal Medicine, Shahjalal University of Science and Technology, Sylhet, BGD; 4 Internal Medicine, Faculty of Medicine, Cairo University, Cairo, EGY; 5 Clinical Research, Sanmora Bespoke Clinical Research Solutions, East Orange, USA; 6 Internal Medicine, California Institute of Behavioral Neurosciences & Psychology, Fairfield, USA

**Keywords:** acute cardiac injury, cardiovascular monitoring protocols, chemo-radio (chemoradiotherapy), chemotherapy-induced cardiotoxicity, chronic cardiac injury, ec- esophageal cancer, radiation dosing, radiation-induced cardiotoxicity

## Abstract

Concurrent chemoradiotherapy (CRT) remains a standard treatment for locally advanced esophageal cancer, yet the approach poses significant cardiotoxic risks due to the proximity of the esophagus to cardiac structures. This systematic review synthesized recent evidence from observational studies and systematic/narrative reviews published in the last five years, with a focus on quantifying the incidence and timing of cardiac events, examining mechanisms of CRT-induced cardiotoxicity, and evaluating strategies for monitoring and mitigating cardiac risk.

A comprehensive search was conducted in PubMed, PubMed Central, Cochrane Library, Google Scholar, and ScienceDirect, restricted to English-language studies published within the past five years, in accordance with Preferred Reporting Items for Systematic Reviews and Meta-Analyses (PRISMA) 2020 guidelines. Eligible studies specifically investigated CRT in esophageal cancer patients and reported cardiac outcomes. Cardiotoxicity is defined as acute (≤90 days), subacute (3-6 months), or chronic (>6 months) outcomes, including pericardial effusion, arrhythmias, ischemic events, myocardial fibrosis, and heart failure, assessed through imaging, biomarkers, or clinical endpoints.

Findings showed that acute events such as pericardial effusion and arrhythmias occur frequently within 90 days post-CRT, while chronic conditions, including ischemic heart disease and heart failure, are more prevalent among long-term survivors. Older patients and those with pre-existing cardiovascular disease were particularly vulnerable to CRT-related cardiac injury.

CRT for esophageal cancer carries substantial acute and chronic cardiotoxic risks. Mechanisms involve radiation-induced fibrosis and endothelial injury, compounded by chemotherapy-associated oxidative stress and vasospasm. Clinical implications include the adoption of heart-sparing radiotherapy techniques, systematic biomarker monitoring (BNP, troponin), and cardioprotective agents such as beta-blockers or ACE inhibitors. Advanced imaging (cardiac MRI, echocardiography) offers opportunities for early detection of subclinical injury. Future research should prioritize standardized dosimetry for cardiac substructures (e.g., mean heart dose, LAD constraints) and prospective studies integrating biomarker- and imaging-based cardiac assessment tools to better balance oncologic efficacy with cardioprotection, ultimately improving survival and quality of life in this high-risk patient population.

## Introduction and background

Esophageal cancer ranks as the eighth most prevalent cancer globally, with around 604,000 new diagnoses and 544,000 deaths each year, but incidence and mortality vary markedly across regions, with higher burdens in East Asia and parts of Africa and rising trends in Western countries linked to adenocarcinoma [1,2]. Although treatment strategies such as multimodal therapy, targeted approaches, and improved surgical techniques have advanced, the overall five-year survival rate for most patients remains under 20%, emphasizing the disease’s aggressive progression [3]. For patients with locally advanced esophageal cancer, chemoradiotherapy (CRT) has become a critical treatment, improving locoregional control and increasing survival by 10-15% compared with radiation alone, though this benefit varies by tumor stage and patient fitness [4,5]. However, CRT introduces substantial risks, particularly cardiotoxicity, which can severely impact long-term health and quality of life for survivors [6]. 

Cardiotoxicity, defined in clinical practice by imaging, biomarker, or functional thresholds such as left ventricular ejection fraction (LVEF) decline or elevated troponin, is of particular concern in esophageal cancer because the proximity of the esophagus to the heart exposes cardiac structures to high radiation doses during CRT, where mechanisms include endothelial injury, microvascular damage, and fibrosis. Such exposure can result in a range of complications, including pericarditis, coronary artery disease, cardiomyopathy, valvular heart disease (particularly aortic valve disease), and heart failure, with some studies reporting event rates as high as 30% among long-term survivors [1,7]. Chemotherapy agents such as anthracyclines and fluoropyrimidines further exacerbate risks by generating oxidative stress, inducing endothelial injury, and triggering coronary vasospasm, with inflammation and autonomic dysfunction also implicated [8,9]. As survival improves due to multimodal treatments, the long-term cardiac risks associated with CRT are increasingly relevant. Radiation-induced heart disease (RIHD) includes a spectrum of complications that can reduce overall survival in esophageal cancer survivors by up to 15% [10,11]. Advanced technologies like intensity-modulated radiotherapy (IMRT) reduce cardiac exposure, though barriers such as cost, access, and technical expertise limit their broader adoption [12]. 

Innovations in imaging and biomarker monitoring, including cardiac MRI-derived extracellular volume (ECV) mapping and biomarkers such as BNP or troponin, show promise for early detection but remain limited by validation, standardization, and clinical uptake [3]. Despite these advances, evidence remains limited, particularly long-term follow-up, consistent dosimetric reporting, and studies addressing predictors and preventive strategies, partly due to challenges in maintaining cohorts, funding, and harmonizing outcome definitions.

This systematic review investigates CRT-induced cardiotoxicity in esophageal cancer, with emphasis on clinical outcomes, mechanistic pathways, and emerging diagnostic innovations. 

## Review

Methods

The Preferred Reporting Items for Systematic Reviews and Meta-Analyses (PRISMA) 2020 criteria served as the basis for this review [13], with explicit attention to search strategy, duplicate removal, eligibility screening, inclusion/exclusion criteria, data extraction by independent reviewers, and risk of bias assessment.

Eligibility of Studies 

The studies were selected based on the Participants, Intervention, and Outcomes (PIO) framework and specific inclusion and exclusion criteria. Participants included patients with esophageal cancer, specifically squamous cell carcinoma or adenocarcinoma, receiving definitive or neoadjuvant CRT. The intervention was defined as concurrent CRT, utilizing chemotherapy agents like cisplatin or 5-FU, and reported radiotherapy (RT) doses and techniques. Outcomes included cardiotoxicity, encompassing both acute and long-term complications, mechanisms of injury, dose-response relationships, diagnostic tools, and potential biomarkers of cardiotoxicity.

Inclusion criteria encompassed studies reporting cardiotoxicity outcomes in esophageal cancer patients undergoing CRT. Randomized controlled trials (RCTs), observational studies, and systematic reviews with or without meta-analyses were among the eligible study designs. The review included only full-text articles published in English within the last five years. Studies involving only human participants were considered, with animal models excluded.

Exclusion criteria included studies on cancers other than esophageal cancer or mixed populations where esophageal cancer outcomes could not be isolated. Studies on RT or chemotherapy alone, non-standard CRT regimens, or those without explicit cardiac outcomes were excluded. Case series, case reports, editorials, non-peer-reviewed articles, conference proceedings, grey literature, and animal studies were not included.

Search Strategy

The search was conducted systematically using PubMed, PubMed Central (PMC), Cochrane Library, Google Scholar, and ScienceDirect databases. The final search date across all databases was October 4, 2024. Keywords and search terms were selected based on previous literature and refined using Medical Subject Headings (MeSH) where applicable, depending on the database as shown in Table 1.

**Table 1:** Database search strategy and results with filtered search and hits. PMC: PubMed Central

Databases	Topic Area	Search Terms	Filters	Hits
Pubmed	Esophageal Cancer, Chemoradiotherapy, Cardiotoxicity	"Esophageal Neoplasms/diagnosis"[Mesh] OR "Esophageal Neoplasms/pathology"[Mesh] OR "Esophageal Neoplasms/physiopathology"[Mesh] OR "Esophageal Neoplasms"[Mesh] OR "Esophageal Cancer" OR "Esophageal Tumors" OR "Malignant Esophageal Lesions" AND ("Chemoradiotherapy"[Mesh] OR "Chemoradiotherapy, Adjuvant"[Mesh] OR "Neoadjuvant Therapy"[Mesh] , "Chemotherapy, Adjuvant"[Mesh] OR "Concurrent Chemoradiotherapy" OR "Combination Therapy" OR "Cisplatin-based Chemoradiotherapy" OR "Carboplatin-based Chemoradiotherapy" OR "Combined Chemoradiotherapy" OR Paclitaxel based chemoradiotherapy OR Capecitabine based chemoradiotherapy OR 5-Fluorouracil based chemoradiotherapy OR Oxaliplatin-based chemoradiotherapy OR Taxane-based chemoradiotherapy, AND "Radiotherapy"[Mesh] AND "radiotherapy" [Subheading] AND "Heavy Ion Radiotherapy"[Mesh] AND "Radiotherapy Setup Errors"[Mesh] AND "Radiotherapy, Image-Guided"[Mesh] AND "Radiotherapy, Intensity-Modulated"[Mesh] AND "Radiotherapy, Conformal"[Mesh] AND "Radiotherapy, Adjuvant"[Mesh] AND "Radiotherapy, High-Energy"[Mesh] AND "Radiotherapy, Computer-Assisted"[Mesh] AND "Radiotherapy Planning, Computer-Assisted"[Mesh] AND "Radiotherapy Dosage"[Mesh] AND "Lymphatic Irradiation"[Mesh] AND "Radiosurgery"[Mesh] AND "Brachytherapy"[Mesh] AND "Radiation Dose Hypofractionation"[Mesh] AND "Dose Fractionation, Radiation"[Mesh] AND "Neoadjuvant Therapy"[Mesh] AND "Cardiotoxicity"[Mesh] OR "Cardiovascular Toxicity" OR "Pericarditis"[Mesh] OR "Radiation-induced Pericarditis" OR "Myocardial Infarction"[Mesh] OR "Heart Attack" OR "Heart Failure"[Mesh] OR "Congestive Heart Failure" OR "Radiation-Induced Heart Disease" OR "Cardiotoxicity"[Mesh]) OR ( "Cardiotoxicity/complications"[Mesh] OR "Cardiotoxicity/epidemiology"[Mesh] OR "Cardiotoxicity/pathology"[Mesh] )	5 years Free-full text English Human trials	147
PMC	Esophageal Cancer, Chemoradiotherapy, Cardiotoxicity	("Esophageal Neoplasms/diagnosis"[Mesh] OR "Esophageal Neoplasms/pathology"[Mesh] OR "Esophageal Neoplasms/physiopathology"[Mesh] OR "Esophageal Neoplasms"[Mesh] OR "Esophageal Cancer" OR "Esophageal Tumors" OR "Malignant Esophageal Lesions") AND ("Chemoradiotherapy"[Mesh] OR "Chemoradiotherapy, Adjuvant"[Mesh] OR "Neoadjuvant Therapy"[Mesh] OR "Chemotherapy, Adjuvant"[Mesh] OR "Concurrent Chemoradiotherapy") AND "Cardiotoxicity"	5 years Open access	79
Cochrane Library	Esophageal cancer, Chemoradiotherapy, Cardiotoxicity	"Esophageal Neoplasms/diagnosis"[Mesh] OR "Esophageal Neoplasms/pathology"[Mesh] OR "Esophageal Neoplasms/physiopathology"[Mesh] OR "Esophageal Neoplasms"[Mesh] OR "Esophageal Cancer" OR "Esophageal Tumors" OR "Malignant Esophageal Lesions" AND "Chemoradiotherapy"[Mesh] OR "Chemoradiotherapy, Adjuvant"[Mesh] OR "Neoadjuvant Therapy"[Mesh] , "Chemotherapy, Adjuvant"[Mesh] OR "Concurrent Chemoradiotherapy" AND "Cardiotoxicity"	25/09/24 to 25/09/19	9
Science Direct	Esophageal cancer, Chemoradiotherapy, Cardiotoxicity	Esophageal Cancer AND Chemoradiotherapy AND Cardiotoxicity	2024-2019 Subject Areas: Medicine and Dentistry, Biochemistry, Genetics and Molecular Biology, Pharmacology, Toxicology and Pharmaceutical Science-	98
Google Scholar	Esophageal cancer, Chemoradiotherapy, Cardiotoxicity	"Esophageal cancer" AND "Chemoradiotherapy" AND "Cardiotoxicity"	2024-2019	557

Evaluation of Data and Outcomes

Due to the variation in demographics, treatment regimens, and outcome measures across studies, a comparative synthesis approach was used, where findings were organized into common themes (incidence, mechanisms, and outcomes) while accounting for heterogeneity. Data were extracted into standardized Excel forms by two independent investigators (CK and HM), capturing study characteristics (author, year, design, population, interventions, outcomes). Any disagreements were resolved using a predefined protocol through discussion, with a third reviewer consulted when consensus could not be reached. 

Timeframes for outcomes followed accepted conventions in CRT-related cardiotoxicity literature, with acute defined as ≤90 days, subacute as 3-6 months, and chronic as >6 months [2,7,9]. Cardiac outcomes were prioritized if they reflected current standards in cardio-oncology, including pericardial effusion, myocardial fibrosis, arrhythmias, ischemia, heart failure, and validated biomarkers (BNP, troponin) or imaging modalities (MRI, echocardiography). 

Dose-response relationships were evaluated based on reported dosimetric parameters (e.g., mean heart dose, LAD V20, pericardial BED), with outcomes compared against these thresholds when available. Categorizing studies by participant group (definitive vs. neoadjuvant CRT) and timing (acute, subacute, chronic) facilitated clearer comparisons and directly addressed the review objective of mapping temporal patterns and mechanisms of CRT-induced cardiotoxicity. 

Quality Assessment Tools

Two independent investigators (CK and BS) reviewed the quality and bias of each included study. In case of a disagreement, a third reviewer finalized the dispute. The Newcastle-Ottawa Scale (NOS) was applied to evaluate cohort and cross-sectional studies (Table 2), while the A Measurement Tool to Assess Systematic Reviews 2 (AMSTAR 2) tool was used for systematic reviews with meta-analyses (Table 3). Narrative reviews were assessed using the Scale for the Assessment of Narrative Review Articles (SANRA) checklist (Table 4) [14-16]. 

**Table 2:** Quality appraisal using the Newcastle-Ottawa Scale Cohort studies: Items 1–4 (Selection Domain): Representativeness of the exposed cohort, selection of the non-exposed cohort, ascertainment of exposure, demonstration that outcome of interest was not present at start of study. Item 5 (Comparability Domain): Comparability of cohorts on the basis of design or analysis. Items 6–8 (Outcome Domain): Assessment of outcome, adequacy of follow-up duration, and adequacy of follow-up of cohorts. Cross-sectional study: Items 1–4 (Selection Domain): Representativeness of the sample, sample size, comparability between respondents and non-respondents, ascertainment of exposure. Items 5–6 (Comparability Domain): Control for confounding factors, control for additional factors. Items 7–8 (Outcome Domain): Assessment of the outcome and appropriateness of statistical tests.

First Author, Year	Item 1	Item 2	Item 3	Item 4	Item 5	Item 6	Item 7	Item 8	Item 9	Quality Score
Cohort Studies										
Jin et al., 2024 [1]	1	0	1	1	1	1	1	1	1	8
Sawyer et al., 2021 [4]	1	0	1	1	1	1	1	1	1	8
Thomas et al., 2019 [5]	1	0	1	1	1	1	1	1	1	8
Celik et al.,2020 [17]	1	1	1	0	1	0	1	0	0	5
Takeuchi et al., 2020 [18]	1	0	1	1	1	1	1	1	1	8
Zhang et al.,2021 [19]	1	0	1	1	1	0	1	1	0	5
Hatayama et al., 2023 [20]	1	0	1	1	1	1	1	1	1	8
Miyoshi et al., 2023 [21]	1	0	1	1	1	1	1	1	1	8
Cross-sectional study										
Beukema et al., 2022 [22]	1	1	1	1	1	1	1	1	1	9

**Table 3:** Included studies assessed using a A Measurement Tool to Assess Systematic Reviews 2 (AMSTAR) 2 Items 1–2: Research questions and inclusion criteria, protocol registration and justification for deviations. Items 3–5: Justification of study design, comprehensive literature search strategy, duplicate study selection. Items 6–8: Duplicate data extraction, list of excluded studies with justification, and detailed description of included studies. Items 9–11: Risk of bias assessment, appropriate statistical methods for meta-analysis, and assessment of risk of bias impact on results. Items 12–13: Interpretation of results considering risk of bias and discussion of heterogeneity. Items 14–16: Investigation of publication bias, reporting of funding sources, and disclosure of conflicts of interest. Scoring: Y - Yes; PY - Partial yes; N – No

First Author, Year	Item 1	Item 2	Item 3	Item 4	Item 5	Item 6	Item 7	Item 8	Item 9	Item 10	Item 11	Item 12	Item 13	Item 14	Item 15	Item 16	Quality Score
Folco et al., 2024 [3]	Y	Y	Y	Y	Y	Y	N	Y	Y	N	Y	Y	Y	Y	Y	Y	14
Lu et al., 2022 [6]	Y	N	Y	Y	Y	Y	N	Y	Y	N	Y	Y	Y	Y	Y	Y	13

**Table 4:** Critical appraisal using the Scale for the Assessment of Narrative Review Articles (SANRA) checklist.

First Author, Year	Justification of the Article’s Importance	Statement of Concrete Aims	Description of the Literature Search	Referencing	Scientific Reasoning	Appropriate Presentation of Data	Quality Score
Vosmik et al., 2020 [2]	2	2	1	2	2	2	11
Ritter et al. 2023 [23]	2	2	1	2	2	2	11

Results

Selection Process and Quality Evaluation

**Figure 1:**
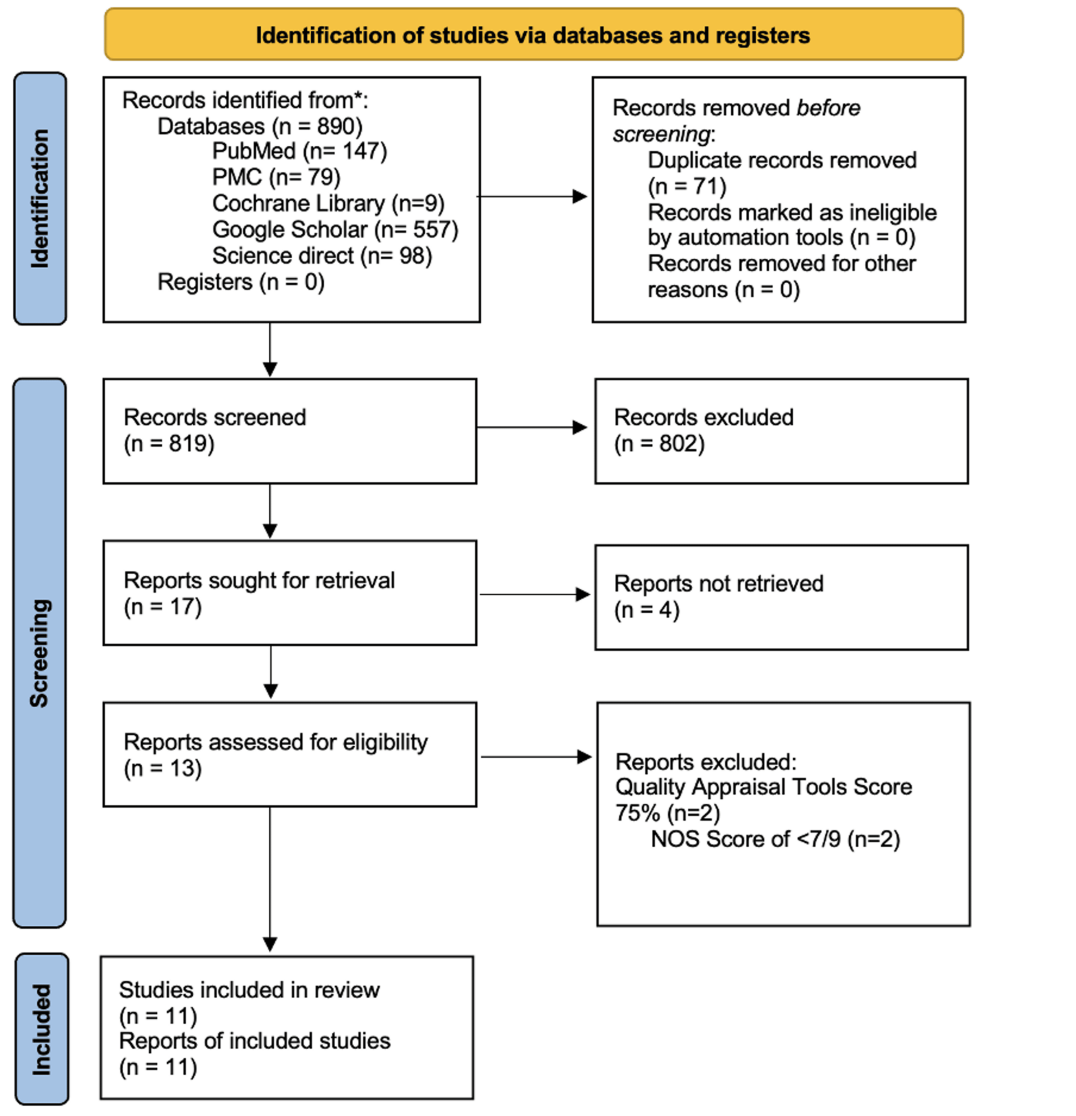
Search selection flow diagram. NOS: Newcastle-Ottawa Scale

The database search initially identified 890 potentially relevant records. Of these, 71 duplicate records were removed, leaving 819 for screening. Titles and abstracts were assessed using the PIO framework and eligibility criteria. Seventeen articles were selected for full-text retrieval; four could not be retrieved. Thirteen studies underwent detailed eligibility assessment, during which quality appraisal was conducted. A 75% threshold was chosen based on prior methodological guidance in systematic reviews, ensuring that only studies with sufficient rigor and low risk of bias were retained. Two studies were excluded for falling below this cutoff, leaving 11 studies. These comprised seven observational studies, two systematic reviews with meta-analysis, and two narrative reviews. Including multiple study types was considered appropriate to comprehensively address the review question, as observational studies provide incidence and dose-response data while systematic/narrative reviews synthesize broader mechanistic and diagnostic insights. The study selection process is illustrated in Figure 1. 

Table 2 shows cohort studies and one cross-sectional study assessed using the NOS [14], with scores of 1 or 0 for each item. The accepted cross-sectional study score was 1 for each item. All the accepted reviews scored 1 for all items except for Item 2. Two studies scored 5 on the NOS but were included because they still provided unique insights into dose-response relationships and cardiac outcomes not captured elsewhere. Their limitations (smaller sample sizes and incomplete follow-up reporting) were acknowledged and considered when interpreting results, preventing them from disproportionately influencing conclusions. Items such as Item 2 (“selection of non-exposed cohort”) consistently scored 0 across multiple studies, reflecting inherent design features in single-arm CRT cohorts rather than bias in evaluation. Consistency in scoring was maintained through independent assessments by two reviewers, with disputes resolved by consensus; although inter-rater reliability was not quantified statistically, calibration exercises were conducted prior to scoring to ensure uniform interpretation. 

Table 3 shows a systematic review with meta-analysis using the AMSTAR 2 tool [15]. Both studies were accepted, with one review having a “NO” in items 7 and 10 and the other having a “NO” in items 2, 7, and 10.

Table 4 shows narrative reviews assessed using the SANRA checklist [16]. Both accepted studies scored “2” across most domains and “1” for Item 3 (“description of the literature search”), reflecting limited transparency in reporting search strategies (e.g., lack of explicit databases searched, search terms, or timeframes). This common weakness may reduce the comprehensiveness of the reviews, but the included studies nonetheless provided useful contextual insights into mechanisms of CRT-related cardiotoxicity. The SANRA scores were therefore used to acknowledge limitations while still justifying inclusion, as these reviews contributed to the broader objective of synthesizing mechanistic and diagnostic perspectives not fully addressed in primary studies. The lower score on Item 3 was noted in interpretation to avoid overstating the robustness of the reviews’ findings. Identical “2” scores across other SANRA domains reflected genuine strengths (clear aims, appropriate referencing, and scientific reasoning) rather than oversights. Scoring consistency was maintained through independent reviewer assessment, with calibration discussions held prior to scoring to standardize interpretations. 

Summary of Cohort and Cross-sectional Studies

The summary of cohort and cross-sectional studies in Table 5 examines cardiac outcomes associated with CRT in esophageal cancer. It includes demographics, CRT interventions, chemotherapy agents, RT techniques, and dosimetric parameters, along with acute, subacute, and chronic complications such as pericardial effusion, myocardial fibrosis, arrhythmias, and heart failure. Insights were provided into treatment-specific factors (pericardial BED, LAD V20, mean heart dose (MHD) thresholds, cisplatin- vs carboplatin-based regimens) and patient-specific factors (age, pre-existing cardiovascular disease, and diabetes). Retrospective cohort studies enabled temporal assessment of incidence and dose response but were limited by confounding, while the cross-sectional study offered detailed imaging-based outcomes without causality, reducing generalizability. Across studies, higher LAD and pericardial doses were associated with increased ischemia and heart failure, elevated pericardial BED correlated with effusion, and advanced age increased the risk of atrial fibrillation. These findings highlight how treatment and patient factors shape cardiac outcomes and emphasize the importance of dose-sparing approaches and close monitoring of high-risk groups. 

**Table 5:** Summary of cohort and cross-sectional studies. CRT: Chemoradiotherapy; 3D-CRT: Three-dimensional conformal radiotherapy; IMRT: Intensity-modulated radiation therapy; VMAT: Volumetric modulated arc therapy; ESCC: Esophageal squamous cell carcinoma; CTV: Clinical target volume; LAD: Left anterior descending artery; V5/V10/V20/V30/V40/V50: Volume of an organ receiving 5, 10, 20, 30, 40, or 50 Gy of radiation; BED: Biologically effective dose; MHD: Mean heart dose; BMI: Body mass index; AF: Atrial fibrillation; PE: Pericardial effusion; BNP: Brain natriuretic peptide; NTCP: Normal tissue complication probability; MRI: Magnetic resonance imaging; CT: Computed tomography; DVH: Dose-volume histogram; ENI: Elective nodal irradiation

First Author, Year	Study Type	Patient Population	Intervention Type	Chemotherapy Agents	Radiotherapy Technique and Dosage	Radiotherapy Target and Dosimetric Parameters	Follow-up Duration	Cardiac Outcome (acute ≤ 90 days, subacute 3–6 months, long-term > 6 months)	Cardiac Risk Factors	Baseline Comorbidities	Cardiotoxicity Assessment Method	Summary of Findings
Jin et al. 2024 [1]	Retrospective Cohort	ESCC, Stage III; n=350; Median age 63 years; 70% male	Definitive Chemoradiotherapy (dCRT)	Carboplatin and Paclitaxel	IMRT with 45-50 Gy (1.8–2 Gy/fx); Proton Therapy for cardiac sparing	LAD V20 <30 Gy, Pericardium; MHD <30 Gy	24 months	Long-term: 30% incidence of ischemia and heart failure, reduced survival	High doses to LAD and pericardium	15% with prior cardiovascular disease	DVH analysis and landmark analysis	LAD dose predictive of major cardiac events (p < 0.05)
Sawyer et al. 2021 [4]	Retrospective Cohort	Stages II–III; n=405 (188 younger: <70 years, 94 older: ≥70 years); Median age 65 years; 68% male	Neoadjuvant CRT and Surgical Resection	Standard Cisplatin-based regimens	Standard CRT protocol, 50.4 Gy in 1.8 Gy/fx	Mediastinal and cervical nodes; V20 <30%	12 months	Long-term: 20% increase in AF in patients ≥70 years	Age (≥70 years)	15% with diabetes or hypertension	Postoperative AF monitoring	Higher rates of AF post-CRT in older patients, comparable survival
Thomas et al. 2019 [5]	Retrospective Cohort	Adenocarcinoma and Squamous Cell Carcinoma, Stages II-III; n=691; Mean age 64 years; 67% male	Preoperative CRT and Surgery	Cisplatin-based, Carboplatin-based, Oxaliplatin-based, Taxane-based	3D-CRT, IMRT, VMAT, Proton Therapy; 36–56 Gy in 1.8–2.0 Gy/fx	Heart and Lung volumes receiving V5–V50 Gy thresholds	18 months	Acute: Cardiac complications in 15% within hospitalization or 30 days	Age, BMI, Cardiac Comorbidity	30% with hypertension or diabetes	NTCP Model	Older age predictive for cardiac events in esophageal cancer undergoing CRT
Takeuchi et al. 2020 [18]	Retrospective Cohort	Stage II-III Esophageal Cancer; n=83; Median age 66 years; 62% male	Definitive CRT	Cisplatin and 5-FU	3D-CRT, 50–71 Gy (1.8–2 Gy/fx)	Heart and pericardium V5–V100-BED	24 months	Long-term: Symptomatic pericardial effusion in 14%	High pericardial BED dose	10% with cardiovascular conditions	BEDVH	High BED dose to pericardium significant risk for symptomatic PE
Hatayama et al. 2023 [20]	Retrospective Cohort	Unresectable Esophageal Cancer, Stage III; n=41; Median age 69 years; 66% male	Definitive Chemoradiotherapy (CRT)	Cisplatin and 5-FU	3D-Conformal RT, 50-60 Gy (1.8–2 Gy/fx)	Heart V5, V10, V20, V30	18 months	Acute-to-Subacute: 34% developed pericardial effusion with BNP changes	High heart doses and BNP ratio changes	Not specified	BNP measurements pre- and post-CRT	Significant BNP changes predictive of cardiac events
Miyoshi et al. 2023 [21]	Retrospective Cohort	Stage I–IV Locally Advanced ESCC; n=65; Median age 70 years; 60% male	Definitive CRT with ENI	Platinum and 5-FU	3D-Conformal RT or IMRT; Median dose 66 Gy (1.8 Gy/fx)	CTV with ENI; V20 <30%, V40 <30%	30 months	Long-term: Grade 5 heart failure observed in 3%	Pre-existing cardiovascular disease	20% with cardiovascular history	Clinical monitoring	Low recurrence but increased cardiac events in high-risk patients
Beukema et al. 2022 [22]	Cross-sectional Study	Stage II–III Esophageal Cancer (CROSS criteria); n=40; Mean age 65 years	Neoadjuvant CRT and Surgery	Carboplatin and Paclitaxel	3D-CRT; 41.4 Gy in 23 fractions (1.8 Gy/fx)	Heart V40 <30%, Lung V20 <30%	36 months	Acute: 15% AF Long-term: 35% myocardial fibrosis,	Baseline cardiopulmonary risk factors	25% with pre-existing pulmonary disease	Cardiac MRI, CT, Echocardiography	Radiation-induced myocardial fibrosis may contribute to AF

Summary of Systematic and Traditional Reviews 

Systematic and narrative reviews addressing CRT-related cardiotoxicity in esophageal and other thoracic malignancies are summarized in Table 6. To aid interpretation, incidence figures are presented with context on follow-up duration, baseline cardiovascular risk, and cardiac dose exposure rather than as isolated point estimates. Some reviews report subgroup modifiers older age, female sex, and pre-existing cardiovascular disease while others do not specify patient-level risks; this variability is indicated to guide risk stratification. Because the table combines systematic/meta-analytic and narrative reviews, the evidence levels differ: systematic reviews provide pooled estimates with greater methodological rigor, whereas narrative reviews contribute mechanistic and contextual insights. Clinically, convergent signals support heart-sparing RT with explicit cardiac constraints, early surveillance using biomarkers and imaging, and individualized monitoring pathways for higher-risk patients. 

**Table 6:** Summary of systematic and traditional reviews. CRT: Chemoradiotherapy; CHF: Congestive heart failure; MHD: Mean heart dose; ECV: Extracellular volume; MRI: Magnetic resonance imaging; V5/V30/V50: Volume of an organ receiving 5, 30, or 50 Gy of radiation; NR: Not reported

First Author, Year	Study Type	Primary Aim	Disease	Intervention	Number and Type of Included Studies	Total Number of Participants	Inclusion & Exclusion Criteria	Cardiac-Specific Outcomes and Key Points	Funding Sources
Vosmik et al. 2020 [2]	Narrative review	To discuss the cardiotoxicity of radiation therapy in esophageal cancer and emphasize heart protection strategies in this context	Esophageal cancer	Radiotherapy only and Chemoradiotherapy (CRT)	Seven studies (Retrospective and Prospective)	78-167 patients	Inclusion: Emphasis on cardiac protection in radiation-treated esophageal cancer patients with specific dose parameter Exclusion: Not specified	Ischemic Heart Disease & Pericardial Effusion: 16.3% incidence of severe events (ischemic heart disease, pericardial effusion) at 5 years; higher heart exposure linked to increased risk. Symptomatic Cardiac Complications: 13.8% incidence of symptomatic radiation-induced complications (pericardial effusion and atrial tachyarrhythmias) at 5 years. Pericardial Effusion: 35.9% at 6 months post-treatment; higher pericardial dose increased symptomatic cases. 27.7% developed pericardial effusion with a mean pericardial dose of 26.1 Gy. Gender-Based Risk: Females had a 4.15 times higher cardiotoxicity risk than males; pericardial effusion was most common. Other Cardiac Complications: 10% experienced symptomatic pericarditis; severe cases included myocardial infarction and heart failure. Overall Cardiotoxicity: 20.4% incidence at 12 months, with higher heart dose parameters in symptomatic cases; women showed elevated risk.	NR
Folco et al. 2024 [3]	Systematic Review and Meta Analysis	To investigate the role of MRI-derived extracellular volume (ECV) as a biomarker for detecting cardiotoxicity from cancer therapy	Primarily breast and esophageal cancer	Chemotherapy only (Anthracycline), Radiotherapy only, Chemotherapy + Targeted Therapy, Chemotherapy + Radiotherapy	Three studies (Prospective)	20-76	Inclusion: Studies on MRI-derived ECV in patients treated with cardiotoxic cancer therapies, with a minimum three-month post-treatment Exclusion: Studies lacking clear treatment regimen, patients with pre-existing cardiac comorbidities and overlapping cohorts.	ECV as a Cardiotoxicity Biomarker: ECV showed significant increases post-CRT, supporting its role as a cardiotoxicity marker. Average ECV Increase: ECV increased by an average of 4.1%, indicating fibrosis-related cardiotoxicity. Myocardial Fibrosis: Significant increase in myocardial fibrosis, with MRI T1 mapping showing a mean ECV increase of 2.5%. Early Fibrosis Markers: Demonstrated myocardial changes with T1 mapping, showing ~3% increase in ECV post-CRT, indicative of early fibrosis.	NR
Lu et al. 2022 [6]	Systematic Review & Meta-Analysis	To systematically assess the incidence and factors influencing fluoropyrimidine-related coronary disorders in cancer patients	Colorectal, esophageal, gastric, and breast cancer	Chemotherapy only (Fluoropyrimidine monotherapy), Chemotherapy combination (Fluoropyrimidine-based combination regimens), Chemoradiotherapy (CRT)	Four studies (Prospective, Retrospective, Case-Control)	46-527 patients per study	Inclusion: Studies on fluoropyrimidine-related coronary disorders with sample size > 20, focusing on solid malignancies Exclusion: Case reports, comments, meeting abstracts, studies with sample size ≤ 20	Coronary Toxicity Incidence: 6.32% incidence of coronary toxicity, with myocardial ischemia as the most frequent event. Angina and Chest Pain: Observed in 5.1% of patients, primarily those with pre-existing cardiac conditions. Coronary Events and Arrhythmias: 8.7% incidence of coronary events, with arrhythmias noted as a common complication. Coronary Complications in High-Risk Groups: Documented coronary complications in 4.77% of patients, highlighting the need for cardiac monitoring in high-risk groups.	NR
Ritter et al. 2023 [23]	Narrative review	To review recent findings on radiation-induced cardiotoxicity, particularly in heart failure from thoracic radiotherapy	Breast, lung, esophageal, and lymphoma	Radiotherapy only, Chemoradiotherapy (CRT)	8 studies (Prospective + Retrospective)	76-571	Inclusion: Studies or data on radiation-induced cardiotoxicity with a particular focus on heart failure and thoracic radiotherapy. Exclusion: No specified	Incidence of Cardiac Events: Congestive heart failure (CHF) rates ranged from 0.2% to 23.6%, with the highest being a 90-day major cardiac event rate of 23.6%. New-Onset Heart Failure: 3.7% rate of new-onset heart failure; 4.2% of patients developed grade 3+ heart failure, associated with dosimetric factors (MHD <15 Gy, V5 <80%, V30 <30%, V50 <7%). Symptomatic Heart Failure in Specific Groups: 0.8% rate of symptomatic heart failure, with higher risk observed in female patients; low CHF incidence of 0.2% recorded in elderly patients. Long-Term Cardiac Mortality: One heart failure-related death recorded 20 months post-CRT, highlighting the need for long-term cardiac monitoring.	NR

Cardiotoxicity Incidence and Types Across Studies

Cardiotoxic event incidence and classification across the included studies are detailed in Table 7 according to acute, subacute, and chronic timing. To improve comparability, incidence is expressed in percentages where reported; qualitative descriptors such as “varies by dose” are standardized as narrative notes with contextual ranges where possible. Confidence intervals and p-values were rarely reported in the primary studies and are therefore not consistently available; this limitation is noted in interpretation. 

**Table 7:** Summary of cardiotoxicity incidence and types across studies. LAD: Left anterior descending artery; ECV: Extracellular volume; CRT: Chemoradiotherapy; NTCP: Normal tissue complication probability; MHD: Mean heart dose; BMI: Body mass index; BED: Biologically effective dose; MRI: Magnetic resonance imaging; CT: Computed tomography

First Author, Year	Cardiac Event Type	Incidence Rate (%)	Timing of Cardiac Events (Acute, Subacute, Chronic)	Associated Risk Factors	Cardiotoxicity Assessment Method
Jin et al. 2024 [1]	Ischemia, Heart Failure	30%	Chronic	High doses to the LAD and pericardium	DVH analysis and landmark analysis
Vosmik et al. 2020 [2]	Ischemic Heart Disease, Pericardial Effusion	16.3% ischemic events, 35.9% pericardial effusion	Subacute	Higher heart exposure	Clinical monitoring and dose analysis
Folco et al. 2024 [3]	Myocardial Fibrosis (ECV)	4.1% increase in ECV	Chronic	Cardiotoxic cancer therapies	MRI-derived ECV, T1 mapping
Sawyer et al. 2021 [4]	Atrial Fibrillation	20% in patients ≥70 year	Sub-acute	Age (≥70 years)	Postoperative Atrial Fibrillation monitoring
Thomas et al. 2019 [5]	Cardiac complications (Acute)	15% within hospitalization or 30 days	Acute	Age, BMI, cardiac comorbidities	NTCP Model
Lu et al. 2022 [6]	Coronary Events, Arrhythmias	6.32% coronary toxicity, 5.1% angina	Acute	Pre-existing cardiac conditions	Clinical monitoring
Takeuchi et al. 2020 [18]	Symptomatic Pericardial Effusion	14%	Chronic	High pericardial BED dose	BEDVH
Hatayama et al. 2023 [20]	Pericardial Effusion, BNP Changes	34%	Acute-to-Subacute	High heart doses, BNP ratio changes	BNP measurements pre- and post-CRT
Miyoshi et al. 2023 [21]	Grade 5 Heart Failure	3%	Chronic	Pre-existing cardiovascular disease	Clinical monitoring
Beukema et al. 2022 [22]	Myocardial Fibrosis, Atrial Fibrillation	35% myocardial fibrosis, 15% AF	Acute and Chronic	Baseline cardiopulmonary risk factors	Cardiac MRI, CT, Echocardiography
Ritter et al. 2023 [23]	Heart Failure	0.2%-23.6% (varies by dose)	Acute and Chronic	High dosimetric factors (MHD <15 Gy)	Clinical records and dosimetric analysis

Risk factors were grouped into treatment-related (radiation dose to LAD, MHD, pericardial BED; chemotherapy regimens) and patient-related (age, baseline cardiovascular function, comorbidities). Older age and pre-existing cardiovascular disease frequently compounded the effects of higher cardiac dose exposure, underscoring interaction between patient- and treatment-specific risks. Timing of outcomes was linked to follow-up periods, with acute pericardial effusion and arrhythmias occurring within 90 days, subacute events such as atrial fibrillation or fibrosis emerging within 3-6 months, and chronic coronary artery disease or heart failure appearing beyond six months. 

Variability across studies reflected differences in population characteristics, staging, and baseline cardiac health, influencing observed incidence rates. More recent studies using advanced RT techniques and closer biomarker/imaging surveillance suggest that cardiotoxicity risks may evolve over time, though long-term mitigation effects remain uncertain. Together, these findings highlight the need for standardized dose-threshold reporting and long-term follow-up to better define the trajectory of CRT-related cardiotoxicity. 

Dose-Response Relationship and Cardiac Risk Table (Radiation-Focused)

Table 8 outlines the dose-response relationships and associated cardiac risks in CRT-treated esophageal cancer, focusing on radiation parameters such as LAD V20, MHD, and pericardial BED. To enhance comparability, the timing of outcomes is explicitly noted as acute (≤90 days), subacute (3-6 months), or chronic (>6 months). 

**Table 8:** Dose-response relationship and cardiac risk table (radiation-focused). LAD: Left anterior descending artery; V20: Volume receiving 20 Gy; MHD: Mean heart dose; BED: Biological effective dose; NTCP: Normal tissue complication probability; CRT: Chemoradiotherapy; IMRT: Intensity-modulated radiation therapy; 3D-CRT: Three-dimensional conformal radiotherapy; AF: Atrial fibrillation

First Author, Year	Radiation Technique	Dose Parameters	Cardiac Outcomes	Dose-Response Observations
Jin et al. 2024 [1]	IMRT with Proton Therapy	LAD V20 < 30 Gy, MHD < 30 Gy; reduced heart dose via proton therapy	Chronic: Ischemia, Heart Failure (30%)	LAD V20 and MHD thresholds correlated with cardiac ischemia and late-stage HF, emphasizing dose management importance
Vosmik et al. 2020 [2]	3D-CRT	Heart V50 > 20 Gy linked to elevated risk of pericardial effusion	Subacute: Pericardial Effusion (16.3%)	Higher exposure above V50 threshold resulted in significantly higher pericardial effusion rates post-CRT
Folco et al., 2024 [3]	3D-Conformal RT	Heart V40, V50; MRI-based ECV for fibrosis assessment	Chronic: Increased ECV (Extracellular Volume) (4.1%)	ECV increase associated with fibrosis progression at V40-V50 doses, linking ECV as a chronic fibrosis indicator
Sawyer et al. 2021 [4]	Standard CRT	V20 < 30% applied to mediastinal and cervical nodes	Sub-acute: AF (20%) in patients >70 years	Dose exposure to V20 correlated with increased AF incidence in older patients, suggesting age as a factor in response
Thomas et al. 2019 [5]	IMRT, VMAT, Proton Therapy	V5-V50; Heart and Pericardium; NTCP model with age and BMI adjustments	Acute: Cardiac Complications (15%) within 30 days	Higher doses in V50 range correlated with early acute complications in patients with higher BMI and age
Lu et al. 2022 [6]	Standard CRT	Heart V20, V30, V40; escalation observed in arrhythmia rates	Acute: Coronary Events; Subacute: Arrhythmias (6.3%)	Higher cumulative dose in V20-V40 range showed significant increase in arrhythmia rates, particularly in high-risk patients
Takeuchi et al. 2020 [18]	3D-Conformal RT	V80-BED for heart/pericardium associated with elevated pericardial risk	Acute: Pericardial effusion (14%); Chronic: Symptomatic PE	V80-BED threshold linked with increased symptomatic PE risk; threshold adjustments may reduce acute pericardial risk
Hatayama et al. 2023 [20]	3D-Conformal RT	V5, V10, V20, V30 for Heart; BNP pre/post-CRT as marker	Acute-Subacute: Pericardial Effusion, BNP elevation (34%)	BNP elevation closely linked with V20-V30 doses, suggesting BNP as a biomarker for early cardiac stress
Miyoshi et al. 2023 [21]	3D-Conformal RT or IMRT	CTV ENI; Median dose 66 Gy; V20 < 30%, V40 < 30% as thresholds	Long-term: Grade 5 Heart Failure (3%)	Median dose of 66 Gy with V20 and V40 thresholds correlated with recurrence and higher late-stage heart failure rates
Beukema et al. 2022 [22]	3D-CRT	Heart V40 < 30%, Lung V20 < 30%; fibrosis threshold analysis	Acute: AF (15%) Subacute: Myocardial fibrosis (35%)	Dose thresholds below V40 for the heart minimized fibrosis risk, indicating protective limit for subacute outcomes
Ritter et al. 2023 [23]	Proton Therapy	Heart V20 < 25% escalation linked to progressive heart failure rates	Acute-Chronic: Heart Failure (0.2%-23%)	Dose escalation above V20 led to progressive HF, highlighting a critical threshold for heart dose management

Definitions of “high-risk” are clarified as patients with pre-existing cardiovascular disease, advanced age (≥70 years), or metabolic comorbidities such as diabetes and hypertension. Biomarker-based studies, such as those using BNP elevation, demonstrated that dose exposure (e.g., heart V20-30) correlated with early subacute cardiac stress and subsequent pericardial effusion, underscoring the potential for biomarkers to serve as early warning tools linked directly to dose parameters. 

Proton therapy, highlighted in several studies, reduced MHD and LAD exposure compared with photon-based IMRT or 3D-CRT, and was associated with lower long-term ischemia and heart failure risk. This contrasts with higher late toxicity observed in photon-based cohorts, supporting proton therapy as a cardioprotective option where accessible. 

The NTCP model integrated age, BMI, and cardiac dose thresholds to predict acute cardiac events, illustrating how modeling can guide individualized treatment planning and balance efficacy with toxicity. 

Overall, these findings demonstrate that dose thresholds such as LAD V20 >20-30 Gy or MHD >30 Gy consistently predict late ischemia and heart failure. In clinical practice, these thresholds argue for stricter dose constraints, preferential use of heart-sparing modalities (including proton therapy), and incorporation of biomarker surveillance into CRT protocols to detect subclinical toxicity and adjust monitoring intensity accordingly. 

Cardiac Complications (Chemotherapy and Combined CRT-Focused)

An overview of cardiac complications linked to chemotherapy and combined CRT in esophageal cancer is presented in Table 9. To standardize interpretation, study populations are described with baseline cardiovascular comorbidities, pre-treatment cardiac assessment (LVEF/ECG/troponin), and key demographics (median age and sex distribution). Chemotherapy exposure is contextualized by regimen, dosing strategy (weekly vs three-weekly; bolus vs continuous infusion), and cumulative dose where reported. Outcomes are categorized as acute, subacute, or chronic, and attribution is specified as predominantly chemotherapy-driven, radiation-related, or an interaction during concurrent CRT. Angina and ischemia are most frequently associated with fluoropyrimidines, whereas arrhythmias and heart failure are more common in the CRT setting with platinum/taxane regimens and higher cardiac doses. These patterns support heart-sparing planning during CRT, modification of high-risk infusion schedules, baseline and on-treatment biomarker/ECG surveillance, and early cardiology co-management for patients with pre-existing cardiovascular disease. Findings should be interpreted cautiously given heterogeneity in dosing and schedules, small sample sizes, and retrospective designs; generalizability is greatest for older, male-predominant, locally advanced disease treated with cisplatin-5-FU or carboplatin-paclitaxel. Future work should prioritize prospective, dose-resolved studies with standardized cardiac endpoints and attribution frameworks.

**Table 9:** Cardiac complications chemotherapy and combined CRT-focused. CRT: Chemoradiotherapy; 5-FU: 5-Fluorouracil; LAD: Left anterior descending artery; ECV: Extracellular volume; MHD: Mean heart dose; BNP: Brain natriuretic peptide; NTCP: Normal tissue complication probability; BED: Biologically effective dose; MRI: Magnetic resonance imaging

First Author, Year	Chemotherapy Agents	Number of Patients (n)	Cardiac Outcomes (timing)	Mechanisms of Cardiotoxicity	Observations and Comments
Jin et al. 2024 [1]	Carboplatin + Paclitaxel	n=350	Chronic: Ischemia, heart failure (30%)	Microvascular injury compounded by radiation exposure	High LAD exposure associated with ischemic events
Vosmik et al. 2020 [2]	Cisplatin + 5-FU	n=350	Subacute: Ischemic heart disease (16.3%), effusion (35.9%)	Endothelial dysfunction and inflammatory response	Dose analysis linked with high radiation exposure
Folco et al. 2024 [3]	Cisplatin, 5-FU	n=120	Chronic: Elevated ECV by 4.1%	Myocardial fibrosis	MRI used for ECV and T1 mapping
Sawyer et al. 2021 [4]	Cisplatin-based regimens	n=405	Subacute: Atrial fibrillation (20%) in older patients (≥70)	Age-related susceptibility to cumulative CRT damage	Higher incidence in older patients suggests age vulnerability
Thomas et al. 2019 [5]	Cisplatin, Carboplatin, Oxaliplatin, Taxane-based	n=691	Acute: Cardiac events (15%) within 30 days post-CRT	Direct myocardial oxidative damage	NTCP modelling identified dose-response relationship
Lu et al. 2022 [6]	Cisplatin + 5-FU	n=405	Acute: Coronary events (6.32%), angina (5.1%)	Coronary vasospasm due to CRT	Monitored clinically
Takeuchi et al. 2020 [18]	Cisplatin + 5-FU	n=83	Chronic: Symptomatic pericardial effusion (14%)	Pericardial fibrosis due to high biologically effective dose (BED)	BEDVH analysis used to evaluate dose relationships
Hatayama et al. 2023 [20]	Cisplatin + 5-FU	n=41	Acute-Subacute: Pericardial effusion (34%)	Endothelial damage and inflammation, BNP elevation	Changes in BNP predictive of cardiac events
Miyoshi et al. 2023 [21]	Cisplatin + 5-FU, Nedaplatin + 5-FU	n=65	Chronic: Heart failure (Grade 5 in 3%)	Oxidative stress leading to myocardial damage	Pre-existing cardiovascular disease increased risk
Beukema et al. 2022 [22]	Carboplatin + Paclitaxel	n=40	Acute: Atrial fibrillation (15%) Chronic: Myocardial fibrosis (35%),	Fibrosis and conduction system interference	Detected by cardiac MRI, highlighting structural effects
Ritter et al. 2023 [23]	Cisplatin + 5-FU	n=100	Chronic: Heart failure progression	Fibrosis, likely cumulative effect of CRT	Heart-sparing techniques applied where possible

Discussion 

This section examines the cardiotoxic effects of concurrent CRT in esophageal cancer, structured by timing of outcomes and mechanisms. CRT, which combines RT with chemotherapy agents such as cisplatin, carboplatin, and 5-FU (5-FU), remains central for locally advanced disease due to its impact on locoregional control and survival. However, the proximity of the esophagus to the heart increases cardiac exposure, while chemotherapy amplifies myocardial stress. Reported complications include pericardial effusion, arrhythmias, coronary artery disease (CAD), and heart failure [2,24,25]. 

The acute, subacute, and chronic complications reported across studies reveal both early-onset and long-term cardiotoxic risks [2,23,26]. Incidence varied, with acute events affecting approximately 15-30% of patients, subacute fibrosis or arrhythmias occurring in 20-35%, and chronic CAD or heart failure developing in 10-30% of long-term survivors. This variability reflects differences in dosimetry, chemotherapy regimens, and patient risk factors. Early monitoring with imaging and biomarkers shows potential for detecting preclinical damage, which may allow more timely intervention [27]. 

Cardiac Complications Post-CRT in Esophageal Cancer Patients 

Analysis of the 11 included studies highlights acute (≤90 days), subacute (3-6 months), and chronic (>6 months) cardiotoxicity. Acute complications frequently included pericardial effusion, ischemia, and arrhythmias. Jin et al. reported that nearly 30% of patients experienced acute ischemic events and heart failure shortly after high-dose CRT, with a left anterior descending artery (LAD) V20 greater than 20 Gy correlating with increased risk [1]. Hatayama et al. observed a 34% incidence of pericardial effusion in the first 90 days after CRT, accompanied by significant rises in B-type natriuretic peptide (BNP) levels as an early biomarker of cardiac stress [20]. Similarly, Miyoshi et al. found that arrhythmias occurred in up to 15% of patients, particularly those treated with higher cisplatin doses above 50 mg/m² per cycle, underscoring the heightened acute risk associated with this agent [21]. These findings highlight the importance of dose-volume metrics such as LAD exposure and MHD in predicting acute cardiac events [28,29]. 

Subacute cardiotoxicity, typically observed within 3-6 months, often involved progressive myocardial damage, inflammatory changes, and early coronary dysfunction. Beukema et al. documented myocardial fibrosis in 35% of patients assessed by cardiac MRI, with significant correlations to an MHD exceeding 30 Gy [22]. Sawyer et al. noted a 20% increase in atrial fibrillation among older patients (≥70 years) within six months of CRT, suggesting that both age and dosimetric exposure modulate vulnerability [4]. Takeuchi et al. further identified early coronary abnormalities in nearly 10% of patients, including impaired coronary flow reserve likely due to oxidative stress and microvascular injury [18]. 

Chronic CRT-related cardiotoxicity encompassed CAD, heart failure, and cardiomyopathy manifesting beyond six months. Thomas et al. demonstrated that patients receiving cumulative high doses, particularly with LAD V20 above 30 Gy, had increased long-term cardiac events [5]. Folco et al. observed persistent myocardial fibrosis in 25% of CRT survivors and diastolic dysfunction in 15% [3]. Ritter et al. emphasized the cumulative impact of CRT, showing progressive functional decline over time, particularly among those with baseline cardiovascular risk factors [23]. Studies using advanced imaging such as MRI and echocardiography highlighted progressive fibrosis and dysfunction, reinforcing the need for long-term cardiac monitoring [30,31]. Overall, early complications such as pericardial effusion and arrhythmias appeared to predispose patients to subsequent chronic dysfunction, with variability in thresholds (MHD >15 Gy versus >30 Gy) reflecting differences in study design and patient populations. 

Mechanisms of CRT-Induced Cardiotoxicity

The mechanisms underlying CRT-induced cardiotoxicity reflect the combined effects of radiation and chemotherapy. Radiation primarily damages cardiac substructures such as the LAD and pericardium. Jin et al. demonstratedthat LAD V20 values above 20 Gy significantly correlated with myocardial ischemia and poorer survival over a 24-month period [1]. Thomas et al. reported that exceeding an MHD of 30 Gy predicted both acute and chronic cardiac events, especially in patients with cardiovascular comorbidities [5]. Ritter et al. observed that higher V20 exposure was associated with endothelial dysfunction and fibrosis, leading to CAD in up to 15% of patients [23]. Beukema et al. identified myocardial fibrosis in 35% of patients with heart V20 above 30%, confirming the dose-dependent nature of late cardiac injury [22]. These results are consistent with experimental evidence showing that oxidative stress and inflammatory cytokines such as IL-6 and TNF-α mediate endothelial injury, ultimately promoting fibrosis [32,33]. 

Chemotherapy exerts complementary and at times synergistic effects. Cisplatin induces mitochondrial damage and generation of reactive oxygen species, with troponin elevation reflecting subclinical myocardial injury [18,34]. 5-FU has been strongly associated with acute coronary vasospasm, with Miyoshi et al. reporting ischemic symptoms in 18% of patients receiving high doses above 800 mg/m², accompanied by troponin elevation [21,35,36]. When administered together with radiation, these agents appear to compound injury. Hatayama et al. noted that 34% of patients developed pericardial effusion during CRT, with cisplatin amplifying the pericardial response [20]. Thomas et al. showed that cisplatin-treated patients exposed to LAD V20 above 30 Gy had a 40% higher risk of heart failure after two years [5]. Folco et al. observed increased cardiac fibrosis and diastolic dysfunction in CRT patients compared to those receiving RT alone [3]. Collectively, these findings suggest that CRT accelerates fibrosis, arrhythmogenesis, and ischemia through oxidative stress, endothelial dysfunction, and inflammation, with additive risks from chemotherapy and radiation exposure. 

Mitigation Strategies and Clinical Implications 

The evidence from these studies underscores the importance of limiting radiation exposure to critical cardiac substructures and tailoring chemotherapy use to minimize cumulative cardiotoxicity. Emerging consensus suggests that LAD V20 should be kept below 20 Gy and MHD below 15 Gy wherever feasible, although thresholds varied across studies [1,22]. Advanced RT delivery techniques such as intensity-modulated RT and proton therapy can reduce cardiac exposure [1,3], yet barriers including cost, accessibility, and technical expertise limit widespread adoption [12]. Parallel to this, biomarkers such as BNP and troponin [20,22], along with advanced imaging modalities like strain echocardiography and cardiac MRI [3,30], have shown promise for early detection of subclinical injury, although their diagnostic thresholds, specificity, and sensitivity in CRT remain insufficiently validated [31]. 

Pharmacological cardioprotection represents another avenue for intervention. Beta-blockers and ACE inhibitors may reduce myocardial oxygen demand, limit ventricular remodeling, and delay the onset of symptomatic heart failure [37]. Statins offer anti-inflammatory and endothelial protective effects, while dexrazoxane has been explored as an adjunct in reducing chemotherapy-induced oxidative injury [38,39]. Prospective studies will be required to confirm whether these strategies translate into improved outcomes in CRT-treated esophageal cancer patients. Together, these findings highlight that integrating dosimetric optimization, cardioprotective therapy, and biomarker-guided surveillance into CRT protocols could enhance both efficacy and safety. 

Limitations

This review has several limitations that warrant consideration. Variation in study populations, CRT regimens, and reporting of cardiac outcomes introduced heterogeneity, reflected in differences such as fibrosis rates ranging from 20 to 35% and variable MHD thresholds (>15-30 Gy). Short follow-up durations in many studies may underestimate chronic outcomes, particularly late-onset fibrosis and heart failure, which often manifest years after treatment. While LAD and pericardial dose parameters were consistently reported, other cardiac substructures, including atria and the right ventricle, remain underexplored. 

The inconsistent use of biomarkers (BNP, troponin) and limited application of advanced modalities such as strain echocardiography or high-sensitivity troponin assays reduce the ability to detect subclinical cardiotoxicity across cohorts. Excluding case reports and grey literature may have limited insights into rare toxicity patterns, though this decision ensured methodological rigor. Similarly, interactions between chemotherapy agents were not uniformly assessed, particularly combinations such as Cisplatin with newer agents. 

Despite these challenges, the studies collectively highlight important patterns, such as higher cardiotoxicity in older patients and those with pre-existing cardiovascular disease, and the influence of dosimetric thresholds on outcomes. Future research should focus on standardized outcome definitions, incorporation of multimodal assessments (imaging, biomarkers, and dosimetry), and longer-term follow-up, ideally extending beyond five years, to capture the full spectrum of CRT-induced cardiotoxicity in esophageal cancer. 

## Conclusions

In conclusion, this systematic review demonstrates that CRT for esophageal cancer is associated with clinically significant cardiotoxicity, with acute manifestations such as pericardial effusion and arrhythmias occurring early, and chronic sequelae including heart failure and myocardial fibrosis emerging over time. These distinct temporal patterns highlight the need for structured, longitudinal cardiac surveillance to reduce morbidity, limit hospitalizations, and preserve long-term quality of life in survivors.

Mechanistically, cardiotoxicity reflects the combined effects of radiation-induced endothelial injury and fibrosis together with chemotherapy-related oxidative stress, mitochondrial dysfunction, and inflammatory signaling, accelerating coronary and myocardial disease. Clinically, risk mitigation should focus on limiting cardiac substructure exposure, such as maintaining LAD V20 <20 Gy and mean heart dose <15 Gy, individualizing treatment based on baseline cardiovascular risk, and integrating advanced radiotherapy techniques, cardioprotective pharmacotherapy, and sensitive biomarker- and imaging-based surveillance. Future research should emphasize personalized CRT strategies, real-time dosimetric monitoring, and longer follow-up, supported by multidisciplinary cardio-oncology collaboration to optimize oncologic efficacy while safeguarding cardiac health.
